# Heterogeneity of Attitudes toward Robots in Healthcare among the Chinese Public: A Latent Profile Analysis

**DOI:** 10.3390/ijerph20010508

**Published:** 2022-12-28

**Authors:** Xuanyi Bi, Yu Gao, Erhong Sun, Yan Yan, Yimin Zhou, Xuchun Ye

**Affiliations:** Department of Nursing, Naval Medical University, Shanghai 200433, China

**Keywords:** robot, attitude, healthcare, cross-sectional study, latent profile analysis, China

## Abstract

Attitudes are deemed critical psychological variables that can determine end users’ acceptance and adoption of robots. This study explored the heterogeneity of the Chinese public’s attitudes toward robots in healthcare and examined demographic characteristics associated with the derived profile membership. The data were collected from a sample of 428 Chinese who participated in an online survey. Latent profile analysis identified three distinct subgroups regarding attitudes toward robots—optimistic (36.9%), neutral (47.2%), and ambivalent (15.9%). Interestingly, although participants in the ambivalent attitude profile held more negative attitudes toward interaction with or social influence of healthcare robots, their attitudes tended to be positive when it came to emotional interactions with healthcare robots. All the respondents reported negative attitudes toward the social influence of healthcare robots. Multivariable regression analysis results showed that there were significant differences in age, education level, monthly income, experience with computers, experience with wearable devices, and whether to follow robot-related news or not. This study confirmed the heterogeneity of the Chinese public’s attitudes toward robots in healthcare and highlighted the importance of emotional interaction with and social influence of healthcare robots, which might facilitate a better understanding of the needs and expectations of potential end users for robots in healthcare to make them more acceptable in different situations.

## 1. Introduction

Over the past few years, populations have been aging at an accelerating pace in numerous countries around the world, with fewer births and more people living alone [1]. The demands for healthcare professionals have also been growing as the size of the aging population increases. Taking effective measures to cope with these changes and reduce the care burden on informal and formal caregivers have gradually become critical issues [2]. New-generation robots have been generally considered valuable solutions for the above social issues, enabling people to enjoy considerable benefits brought by this innovative technology [3,4]. These benefits in the healthcare contexts may be sorted into two main categories: functional assistance and social assistance [5]. On the one hand, robots can perform repetitive and dangerous tasks previously handled by humans [3], deliver effective assistance in activities of daily living (e.g., consuming meals, daily toileting, and doing housework) [4], provide rehabilitation or assistance with upper or lower extremity movements [6], and monitor a user’s health status and physiological parameters [5]. On the other hand, robots may reduce the user’s loneliness and isolation [7], enhance social participation and engagement [8], and improve overall mood and mental health [9]. In China, both commercial and research development of robots have undergone exponential growth in response to an increasingly aging population in recent years [10]. Given the soaring number of robots in daily life and healthcare, a thorough understanding of end users’ needs and expectations for robotic devices in healthcare can be helpful for the design and development of robots in healthcare that could be tailored to the different attitudes toward robots in this context.

The successful adoption of novel technologies is determined by multiple individual factors, such as attitude, effort expectancy, performance expectancy, subjective norms, and prior experience [11,12]. Attitudes toward robots are generally understood to be an extremely persistent personal trait and have become an attractive topic area for researchers in robotics, psychology, and engineering in the past decade. Previous studies indicated that attitudes toward robots could predict the users’ expectations and intention to use during human–robot interaction (HRI), which might facilitate the integration of robots into everyday life or healthcare [13,14]. However, several systematic or scoping reviews have shown a vague picture of people’s attitudes toward robots, making it difficult to conclude whether people hold positive or negative views on the use of robots [15,16,17]. For instance, Chu et al. found that older adults reported positive attitudes toward the use of robots in an aged-care setting [18]. Older adults in the study by Backonja et al. had neutral attitudes toward robots [19], while those in the study by Wu et al. were afraid of having assistive robots in their daily life [20]. These discrepancies needed further exploration. 

In fact, recent studies have demonstrated that people’s attitudes toward the targeted object may be ambivalent and cannot be simply described as just negative or positive [21], which have been studied in various domains, such as artificial intelligence (AI) [22] and online private-information sharing [23]. In the healthcare context, a qualitative meta-synthesis revealed the ambivalent nature of end users’ attitudes toward robotics in motor rehabilitation, namely the dilemma between benefits (e.g., improvement in activities of daily life) and challenges (e.g., the fear of being controlled by robots) during the interaction with robotic devices [24]. In addition, several quantitative studies explored the relationship between ambivalent attitudes toward robots and other key variables through experiments, such as their mental abilities [13] and autonomy [21]. Therefore, it seems crucial to identify distinct clusters of attitudes toward robots in healthcare within a homogeneous population of interest, which will provide empirical insights into the development and potential future uses of healthcare robots in HRI research.

Despite the rapid development and wide use of robots in the healthcare industry, current research seems to be far from sufficient and conclusive. A very limited number of relevant articles have been found to explore the heterogeneity of attitudes toward robots using latent profile analysis (LPA) [14,22,25], which is a person-centered statistical approach that can categorize individuals into different hidden subgroups based on their responses to observed variables [26]. For instance, Manzi et al. explored unobserved subgroups of expectations about future robots among Italian emerging adults aged 18–29 years and identified the following five profiles: mechanical properties, fuzzy, human attributes, human relationship, and self-determination [14]. Bao et al. segmented the U.S. population into distinct profiles of public attitudes toward AI based on their risk and benefit perception and revealed five classes, namely the negative, the ambivalent, the tepid, the ambiguous, and the indifferent [22]. Papadakis et al. detected two clusters of attitudes toward the use of educational robotics (ER) among early childhood teachers, namely “for the use of ER” and “against the use of ER” [25]. These above studies analyzed the different profiles of attitudes toward robots among emerging adults, the public, and teachers. However, as far as we know, no existing studies have been found to explore the heterogeneity of attitudes toward robots in healthcare among the public.

Hence, the main purpose of this study was to identify the distinct profiles of attitudes toward robots in healthcare among the public and examine the demographic characteristics associated with these profiles, which will fill a significant gap in the literature regarding the heterogeneity of attitudes toward robots in healthcare. The research questions are listed as follows: (1) Which profiles can be identified among the Chinese public regarding their attitudes toward robots in healthcare, and what are the features of these profiles? (2) How do participants in each profile differ in their demographic characteristics?

## 2. Materials and Methods

### 2.1. Study Design and Setting

This online cross-sectional survey study was conducted over three months from March to June 2022 including a convenience sample of the Chinese public. The study procedures are prepared and reported according to the Strengthening the Reporting of Observational Studies in Epidemiology (STROBE) Checklist [27] and the Checklist for Reporting Results of Internet E-Surveys (CHERRIES) [28]. Ethical approval for this study was obtained from the Human Research Ethics Committee of the Naval Medical University (2020-GZR-HS-003).

### 2.2. Study Participants

Participants were eligible for the study who met the following inclusion criteria: 18 years or older, reported having access to mobile phones for personal use, being able to understand the written Chinese language, providing informed consent, and volunteering for this study. Respondents were excluded who were identified as having completed relevant questionnaires previously or reported a history of cognitive impairment or psychiatric disorders.

### 2.3. Measures and Data Collection

#### 2.3.1. Questionnaire

*Attitudes toward robots* were assessed with the 14-item Negative Attitudes toward Robots Scale (NARS), which was originally developed in Japan based on theoretical models about computer anxiety and communication apprehension [29,30]. The NARS had been well-validated and widely used in multiple contexts, comprising three domains regarding different aspects of attitudes toward robots (interaction, social influence, and emotional interaction) [29,30]. Participants reported their level of agreement with each item on a five-point scale from 1 (completely disagree) to 5 (completely agree), with higher scores indicating more negative attitudes toward robots. The NARS has been translated into Chinese and validated by this research group and contained three dimensions that measure negative attitudes toward: interaction with robots (NARS-S1) with 6 items, social influence of robots (NARS-S2) with 5 items, and emotional interactions with robots (NARS-S3) with 3 items. In this study, the NARS had a good internal consistency reliability (Cronbach’s alpha = 0.85). In addition, self-reported items were used to collect respondents’ demographic characteristics, including gender, age, education level, monthly income, chronic diseases or not, experience with healthcare robots, experience with computers, experience with wearable devices, experience with automatic devices (e.g., vending machines and automated teller machines), and whether they follow robot-related news or not.

#### 2.3.2. Data Collection

All participants were invited to complete a set of questionnaires through an online survey platform in Mainland China called Wenjuanxing (www.wjx.cn, accessed on 9 July 2022), on which all the items needed to be completed before submission. A recruitment poster, including the following sections: aim; procedure; consent for voluntary participation; and the declaration of anonymity, privacy and confidentiality, was sent to selected individuals or groups via the WeChat app (Version 8.0.31), the most popular social media platform in China. Those interested individuals could then complete the questionnaire and submit it online by scanning the QR code or clicking on the e-link. In this online survey, informed consent was considered to have been obtained once participants clicked the “answering the survey” button as their response to the recruitment poster. 

### 2.4. Data Analysis

Exploratory latent profile analysis (LPA) was performed to identify the unobserved classes with similar characteristics based on the responses on the 14 items of the NARS, using the maximum likelihood method [31]. Fit indices of measurement models were examined, starting with one profile initially and adding profiles incrementally until the exploratory power could not be increased. The model with the best data fit was determined according to statistical criteria, model parsimony, and clinical interpretability. Standard model fit indices included the Akaike information criterion (AIC), Bayesian information criterion (BIC), sample-size-adjusted Bayesian information criterion (aBIC), Lo–Mendell–Rubin likelihood ratio test (LMR-LRT), bootstrapped likelihood ratio test (BLRT), and entropy value. Overall, lower values on AIC, BIC, and aBIC suggested a better model fit, and significant *p*-values (i.e., *p* < 0.05) for the LMR-LRT and BLRT indicated a better model fit for the *k* profile model than the *k* − 1 profile model. The entropy value was used to measure the precision of classification and ranged from 0 to 1, with a higher score indicating greater accuracy [32]. After the optimal model was identified, the profiles would be plotted for each class using a line chart. 

The demographic characteristics of participants were described as means and standard deviations or frequencies and percentages. Once the optimal latent profile model had been identified, chi-square tests were performed to explore demographic characteristics with significant differences among the latent profiles, and the significant variables were entered into multinomial logistic regression analysis, which was conducted to examine the demographic characteristics related to class membership. The above statistical analyses were conducted using IBM SPSS version 24, except LPA, which was performed by MPlus version 8.3. 

## 3. Results

### 3.1. Sample Characteristics

Overall, a total of 446 participants completed the survey, of which 11 insincere responses and 7 duplicate ones were excluded, resulting in 428 responses for the analysis. Of these participants, 54.2% were female (232/428), and the ages ranged from 18 years to 74 years (M = 37.44 years, *SD* = 17.42 years). The detailed demographic characteristics of the sample are listed in Table 1. 

### 3.2. Classification with Latent Profile Analysis

**Figure 1:**
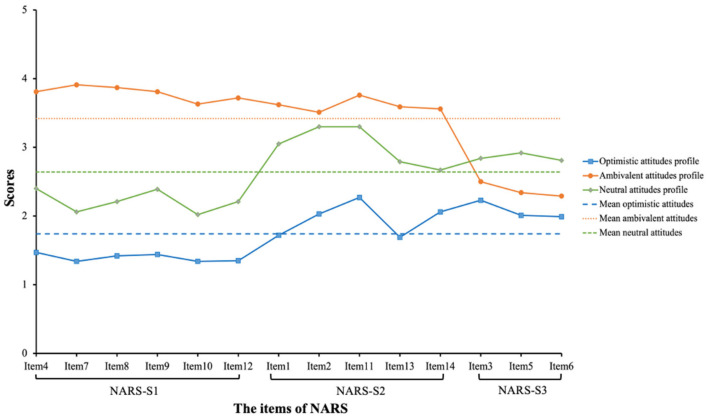
Latent profiles for the three-profile solution.

Four models were estimated via LPA, and the results are presented in Table 2. The AIC, BIC, and aBIC values kept declining from the one-profile model to the three-profile model, while a significant decline in BIC and aBIC values were found between the two-profile model and three-profile model. Namely, the drop in BIC and aBIC values became more gradual from the four-profile model. As the LMR-LRT value (*p* = 0.11) was not significant in the four-profile model, the first three-profile models were compared. Compared with the two-profile model, the three-profile model had lower information criterion indices (AIC, BIC, and aBIC) and all significant LR tests (LMR-LRT *p* = 0.002 and BLRT *p* < 0.001), indicating that the two-profile model should be rejected in favor of the three profiles. Furthermore, the entropy value in the three-profile model was 0.898, denoting the good quality of profile classification. Based on the above results, therefore, the three-profile model was identified as the optimal one in our study. The classification was credible because the attribution probabilities of the three-profile model were 0.940, 0.950, and 0.978, respectively. Figure 1 graphically depicted the results of the three-profile model.

As shown in Figure 1, the three profiles differed in terms of attitudes toward healthcare robots. Specifically, Profile 1 (N = 158, 36.9%) had the lowest scores for all items of the NARS (mean item scores < 2.27), and this group was labeled as the optimistic attitudes profile. Nearly half of the respondents were categorized into Profile 2 (*N* = 202, 47.2%) characterized by the medium scores across all 14 items of the NARS (range 2.02–3.30), and this profile was labeled as the neutral attitudes profile. Profile 3, accounting for 15.9% (*N* = 68) of the sample, was more complicated than the first two profiles. Participants in this profile reported the highest scores on the NARS-S1 and NARS-S2 (mean item scores > 3.50) but with the mean scores on the NARS-S3 items significantly lower than 3 (range 2.29–2.50). Therefore, the third profile was defined as the ambivalent attitudes profile. The NARS scores of the three profiles were listed in Table 3.

When comparing different profiles with respect to the mean scores for each domain of the NARS, Profile 3 showed more negative attitudes toward interaction with healthcare robots (NARS-S1), while Profiles 1 and 2 expressed more positive scores in comparison to the other two dimensions. Regarding NARS-S2, the mean scores were all above average in the three distinct profiles, indicating that all participants had relatively negative attitudes toward the social influence of healthcare robots. Furthermore, participants in Profile 1 expressed the most positive attitudes toward emotional interaction with healthcare robots (NARS-S3), followed by Profile 3 and Profile 2, respectively. Interestingly, although participants in Profile 3 had the highest mean scores on the NARS-S1 and NARS-S2, the mean scores on the NARS-S3 dropped dramatically, suggesting that despite their most negative attitudes toward interaction with or social influence of healthcare robots, their attitudes appeared to be positive when it referred to emotional interactions with healthcare robots.

### 3.3. Demographic Characteristics Associated with Profile Membership

Once the optimal latent profile model was identified, chi-square tests were performed to investigate potential predictors for profile membership. We found significant differences in age, education level, monthly income, chronic diseases or not, experience with computers, experience with wearable devices, experience with automatic devices, and whether they follow robot-related news or not, which would be involved in the following multivariable regression analysis. Differences in demographic characteristics among the three profiles are shown in Table 1.

Profile 1 (optimistic attitudes profile) was selected as the reference profile for multinomial logistic regression. As shown in Table 4, compared with Profile 1 (optimistic attitudes profile), a significant negative effect in Profile 2 (neutral attitudes profile) was found for monthly income lower than 5000 CNY (OR = 0.420, 95% CI 0.206–0.857), while significant positive effects were found for an education level of junior high school or below (OR = 9.211, 95% CI 2.603–32.594) or high school (OR = 4.599, 95% CI 1.388–15.239), or junior college (OR = 3.967, 95% CI 1.717–9.166), experience with computers (OR = 3.516, 95% CI 1.260–9.811), and never (OR = 5.176, 95% CI 1.823–14.695) or sometimes (OR = 6.275, 95% CI 3.262–12.068) following robot-related news. Furthermore, a significant positive effect in Profile 3 (ambivalent attitudes profile) was found for an education level of junior high school or below (OR = 31.198, 95% CI 7.547–128.971) or high school (OR = 11.378, 95% CI 2.638–49.074), while significant negative effects were found for being under 45 years old (OR = 0.158, 95% CI 0.045–0.551) or between 45 and 59 years old (OR = 0.210, 95% CI 0.058–0.758), and experience with wearable devices (OR = 0.208, 95% CI 0.083–0.523), compared with Profile 1 (optimistic attitudes profile).

## 4. Discussion

This study provided a critical first step toward investigating the public’s attitudes toward robots in healthcare from an individual-centered perspective. The identification of three distinct profiles showed that individuals did not function as a monolith with respect to their attitudes toward robots in healthcare. In other words, they cannot be dichotomously classified as considering robots “useful” or “useless”. Moreover, the identification of multiple profiles in the current study might facilitate a better understanding of the needs and expectations of potential end users for robots in healthcare to make them more acceptable in different contexts.

### 4.1. Multiple Profiles of Attitudes toward Robots in Healthcare

As expected, three distinct subgroups regarding attitudes toward robots in healthcare were identified in this study, suggesting that these participants had either optimistic, neutral, or ambivalent attitudes toward robots in healthcare. Firstly, respondents in Profile 1 (optimistic attitudes profile) reported the lowest scores on all three NARS dimensions, indicating that participants in this profile had the most positive attitudes toward robots in healthcare. Secondly, almost half (47.2%) of participants in Profile 2 (neutral attitudes profile) scored moderately on all 14 items of the NARS, reflecting their neutral attitudes toward robots. This might be because the vast majority (75.9%) of participants in this study had no actual experience with robots in healthcare, and thus their attitudes toward robots in healthcare could not be effectively evaluated.

Interestingly, in Profile 3 (ambivalent attitudes profile), a limited number (15.9%) of respondents reported lower mean scores on the NARS-S3 than those on the first two dimensions, which had the highest scores across the three profiles. This finding suggested that, even though these respondents reported more negative attitudes toward interaction with or social influence of healthcare robots, their attitudes would shift in a more positive direction when it came to emotional interactions with healthcare robots. Indeed, recent research on social robots showed that social interaction with robots was structured by two fundamental dimensions: warmth and competence [33], which might influence people’s intention to interact with robots [34]. The warmth dimension referred to “traits associated with the perceived intent of robots, such as sincerity, helpfulness, friendliness, and trustworthiness”, while competence embraced “traits related to the perceived ability of robots, such as skill, efficacy, intelligence, and creativity” [33]. These dimensions might provide a theoretical justification for the interpretation of the ambivalent-attitudes profile identified in this study. In detail, respondents in the ambivalent-attitudes profile might be interested in the development of robots with emotional skills, human attributes, and companionship capabilities that would facilitate robots to become full-fledged social partners [14]. Nevertheless, these individuals also believed that the capabilities and social influence of robotic devices in healthcare might not meet their needs and expectations, which would exert significant effects on their perceived safety and acceptance of robots [35].

Across all subgroups, participants had more negative attitudes toward the social influence of healthcare robots (NARS-S2) than in the other two dimensions. Of all items of NARS, item 11 (I feel that if I depend on robots too much, something bad might happen) had the highest mean scores, followed by item 2 (Something bad might happen if robots developed into living beings). This might be explained by Mori’s theory of the uncanny valley, that robots become more appealing as more human attributes appear, while human affinity to robots would decrease into a sense of unease, strangeness, and fear when the anthropomorphism of robots rises to a certain point [36]. In this study, all participants were concerned that robots might have negative impacts on social norms, especially when people relied on robots too much and robots developed into living beings.

### 4.2. The Predictive Effects of Demographic Characteristics on Profile Membership

The focus on the predictive role of demographic characteristics on profile membership among the public was important to understand the different needs and expectations for healthcare robots and provide recommendations for further design and improvement. Compared with the optimistic-attitudes profile, participants with lower education levels, higher monthly incomes, experience with computers, and who never or sometimes follow robot-related news were more likely to be in the neutral-attitudes profile, while those with older age, lower education levels, and no experience with wearable devices showed significantly higher odds of being classified in the ambivalent-attitudes profile.

In this study, participants older than 60 years were more likely to be in the ambivalent-attitudes profile. The association between age and attitudes toward robots had been reported in previous studies. In other words, older adults who entered the gray market showed more negative attitudes toward robots because they thought that novel technologies were far too complicated to operate and control [2,37]. The results showed that lower education levels (junior college or below) were significantly associated with ambivalent and neutral attitudes toward healthcare robots, which was in accordance with previous findings that participants’ overall attitudes toward robots would improve with growth in their education levels [2,38]. In addition, participants with higher monthly incomes had 0.420 times the odds of belonging to the optimistic-attitudes profile compared with the neutral-attitudes profile. This result was not in line with previous studies in which people’s negative attitudes toward robots in healthcare were associated with low monthly incomes [39]. This might be explained by almost half (47.0%) of participants with lower monthly incomes in this study being college students who tended to express more positive attitudes toward robots in healthcare because of their familiarity and competence with novel technologies [40].

Compared with the optimistic-attitudes profile, participants who never or sometimes followed robot-related news were more likely to be classified in the neutral-attitudes profile. In recent years, the frequent coverage of robot-related news in the Chinese media and the widespread dissemination of information about robots, as well as their effective use in public services and everyday life might gradually increase the awareness of these novel technologies among the public [14,41]. Furthermore, this study also found that experience with computers and wearable devices had statistically significant predictive effects on participants’ attitudes toward robots in healthcare. Compared with the optimistic-attitudes profile, participants without experience with wearable devices were more likely to be in the ambivalent-attitudes profile, whereas those with computer experience had a higher likelihood of being in the neutral-attitudes profile. As we all know, many families were equipped with computers for work, study, or entertainment, while the use of wearable devices could be determined based on their personal interests and actual needs [42]. Like wearable devices, healthcare robots could act in the roles of caregivers and companions in nursing and daily life [7], but they were not essential and could be selected according to the needs and expectations of end users. Therefore, participants who had experience with wearable devices expressed more positive attitudes toward robots in healthcare.

### 4.3. Limitations and Future Research

Nevertheless, the current study suffers from three limitations. First, the first impression of robots was surveyed in the current study, but attitudes and perceptions might change with interactions with robots [43]. The cross-sectional design might hinder speculation on the trajectory of attitudes toward robots. Longitudinal studies may be conducted in the future to explore latent transitions in profile membership between different time points, using latent transition analysis (LTA). Second, an online survey was conducted for data collection and was thus vulnerable to the flaws of this method. Participants were invited to complete electronic questionnaires voluntarily upon placing recruitment posters on the WeChat app, and such non-random sampling methods may evoke sample bias. For example, residents who lack access to the Internet and are unable to speak or read Mandarin might be excluded from this survey. The results should be treated with caution due to the limited representativeness. Hence, a combination of paper and electronic questionnaires might be adopted to address this limitation in future studies. Moreover, the examination of variations among the public appears to be limited because of the relatively small sample size and the relatively imbalanced sample structure, but some interesting findings were still revealed in this study. More diverse and larger samples should be recruited for further studies to allow researchers to explore differences in attitudes toward robots among participants with different characteristics.

### 4.4. Implications

Despite the above limitations, the study still has several significant valuable implications. First, end users should be involved in the design and development of future robots in healthcare to account for the various perspectives held by different subgroups regarding interaction with and the social influence of healthcare robots. User-centered design is an iterative process that involves designers, researchers, and stakeholders in the development process to interpret an emerging design together [44], which has been demonstrated as increasingly important in HRI research [45,46]. Hence, user-centered design approaches should be applied to actively involve end users in the design process and explore their needs, attitudes, and expectations for robots in healthcare, which will prompt the development of robots in healthcare that are more acceptable to end users.

Second, our findings highlight the importance of focusing on the social influence of robots in the design process, which would be critical to promoting end users’ willingness to integrate robots into their daily life and work [47]. Specifically, all respondents reported negative attitudes toward the social influence of healthcare robots, indicating that they might be disproportionately influenced by the social, ethical, and legal concerns about some existing robots in healthcare. Thus, future robots should be designed with a focus on controlling their potential risks (e.g., social, financial, psychological), so that end users’ concerns might be reduced or eliminated during interaction with healthcare robots.

Finally, as people age, there is a growing need for socio-emotional support for the elderly, and healthcare robots have been shown to be a potential approach to meet this need [48]. The study shows that participants in the ambivalent-attitudes profile, particularly older adults, expect emotional interactions with healthcare robots despite their potential dissatisfaction with the functions or social influence of existing healthcare robots, which provides some detailed insights into future avenues of HRI research. Therefore, the dynamic needs and expectations of specific end users for emotional interaction with healthcare robots should be understood by designers, researchers, and engineers in the design and development process.

## 5. Conclusions

The study confirms the heterogeneity of attitudes toward robots in healthcare among the public, which is mainly reflected as optimistic, neutral, and ambivalent attitudes, and fills the gap in HRI-related research. Several demographic characteristics have been found to predict profile membership, including age, education level, monthly income, experience with computers, experience with wearable devices, and whether they follow robot-related news or not. Based on these results, this study might provide useful information for designing and developing robots in healthcare to make them more acceptable in multiple settings according to the needs and expectations of potential end users, especially their emotional interaction with and social influence of healthcare robots. Furthermore, our findings should be re-examined in other contexts and populations, especially typical healthcare customers, to increase their generalizability in HRI research.

## Figures and Tables

**Table 1:** Demographic characteristics of the three profiles.

Variables	Categories	Total *n* (%)	Profile 1 (*N* = 158) *n* (%)	Profile 2 (*N* = 202) *n* (%)	Profile 3 (*N* = 68) *n* (%)	*x*^2^ (*p*)
Gender	Male	196 (45.8)	80 (50.6)	88 (43.6)	28 (41.2)	2.479 (0.290)
	Female	232 (54.2)	78 (49.4)	114 (56.4)	40(58.8)	
Age	<45 years old	258 (60.3)	138 (87.3)	98 (48.5)	22 (32.4)	114.311 (<0.001)
	45–59 years old	109 (25.5)	14 (8.9)	78 (38.6)	17 (25.0)	
	≥60 years old	61 (14.2)	6 (3.8)	26 (12.9)	29 (42.6)	
Education level	Junior high school or below	69 (16.1)	6 (3.8)	32 (15.8)	31 (45.6)	131.639 (<0.001)
	High school	48 (11.2)	5 (3.2)	28 (13.9)	15 (22.1)	
	Junior college	69 (16.1)	11 (7.0)	50 (24.8)	8 (11.8)	
	Bachelor’s degree or above	242 (56.6)	136 (86.0)	92 (56.5)	14 (20.5)	
Monthly income	≤5000 CNY (<$745)	324 (75.7)	138 (87.3)	132 (65.3)	54 (79.4)	23.922 (<0.001)
	>5000 CNY (>$745)	104 (24.3)	20 (12.7)	70 (34.7)	14 (20.6)	
Chronic diseases or not	Yes	86 (20.1)	19 (12.0)	51 (25.2)	16 (23.5)	10.248 (0.006)
	No	342 (79.9)	139 (88.0)	151 (74.8)	52 (76.5)	
Experience with healthcare robots	Yes	103 (24.1)	46 (29.1)	41 (20.3)	16 (23.5)	3.784 (0.151)
	No	325 (75.9)	112 (70.9)	161 (79.7)	52 (76.5)	
Experience with computers	Yes	352 (82.2)	140 (88.6)	172 (85.1)	40 (58.8)	31.089 (<0.001)
	No	76 (17.8)	18 (11.4)	30 (14.9)	28 (41.2)	
Experience with wearable devices	Yes	197 (46.0)	99 (62.7)	89 (44.1)	9 (13.2)	47.341 (<0.001)
	No	231 (54.0)	59 (37.3)	113 (55.9)	59 (86.8)	
Experience with automatic devices	Yes	323 (75.5)	131 (82.9)	160 (79.2)	32 (47.1)	35.897 (<0.001)
	No	105 (24.5)	27 (17.1)	42 (20.8)	36 (52.9)	
Whether they follow robot-related news or not	Never	47 (11.0)	12 (7.6)	25 (12.4)	10 (14.7)	59.527 (<0.001)
	Sometimes	283 (66.1)	78 (49.4)	158 (78.2)	47 (69.1)	
	Always	98 (22.9)	68 (43.0)	19 (9.4)	11 (16.2)	

**Table 2:** Fit indices for models with increasing numbers of latent profiles.

Model	Model Fit Indices						Latent Profile Distribution Rate (%)			
	AIC	BIC	SABIC	Entropy	LMR	BLRT	1	2	3	4
1 profile	18,152.432	18,266.087	18,177.232	na	na	na	100			
2 profile	16,666.040	16,840.582	16,704.127	0.954	<0.001	<0.001	80.1	19.9		
3 profile	15,871.912	16,107.342	15,923.285	0.898	0.002	<0.001	36.9	47.2	15.9	
4 profile	15,674.893	15,971.209	15,739.551	0.914	0.110	<0.001	35.3	47.4	14.0	3.3

Note: AIC = Akaike information criterion; BIC = Bayesian information criterion; SABIC = sample-size-adjusted BIC; LMR = Lo–Mendell–Rubin likelihood ratio test; BLRT = bootstrapped likelihood ratio test; na = not applicable.

**Table 3:** NARS scores of the three profiles.

Domains	Items	Total (*N* = 446)	Profile 1 (*N* = 158)	Profile 2 (*N* = 202)	Profile 3 (*N* = 68)
NARS-S1	4. I would feel uneasy if I was given a job where I had to use robots.	2.28 ± 1.09	1.47 ± 0.57	2.40 ± 0.85	3.81 ± 0.83
	7. The word “robot” means nothing to me.	2.09 ± 1.06	1.34 ± 0.53	2.06 ± 0.62	3.91 ± 0.81
	8. I would feel nervous operating a robot in front of other people.	2.18 ± 1.07	1.42 ± 0.58	2.21 ± 0.73	3.87 ± 0.81
	9. I would hate the idea that robots or artificial intelligences were making judgments about things.	2.26 ± 1.06	1.44 ± 0.59	2.39 ± 0.75	3.81 ± 0.78
	10. I would feel very nervous just standing in front of a robot.	2.03 ± 1.02	1.34 ± 0.47	2.02 ± 0.69	3.63 ± 0.95
	12. I would feel paranoid talking with a robot.	2.13 ± 1.04	1.35 ± 0.53	2.21 ± 0.71	3.72 ± 0.88
NARS-S2	1. I would feel uneasy if robots really had emotions.	2.65 ± 1.19	1.72 ± 0.82	3.05 ± 1.00	3.62 ± 0.96
	2. Something bad might happen if robots developed into living beings.	2.86 ± 1.24	2.03 ± 1.08	3.30 ± 1.01	3.51 ± 1.14
	11. I feel that if I depend on robots too much, something bad might happen.	2.99 ± 1.27	2.27 ± 1.30	3.30 ± 1.01	3.76 ± 1.02
	13. I am concerned that robots would be a bad influence on children.	2.51 ± 1.09	1.69 ± 0.78	2.79 ± 0.87	3.59 ± 0.93
	14. I feel that in the future society will be dominated by robots.	2.59 ± 1.15	2.06 ± 1.10	2.67 ± 0.98	3.56 ± 1.06
NARS-S3	3. I would feel relaxed talking with robots.	2.56 ± 1.04	2.23 ± 1.13	2.84 ± 0.89	2.50 ± 1.00
	5. If robots had emotions, I would be able to make friends with them.	2.49 ± 1.05	2.01 ± 0.95	2.92 ± 0.94	2.34 ± 1.06
	6. I feel comforted being with robots that have emotions.	2.43 ± 1.02	1.99 ± 0.91	2.81 ± 0.97	2.29 ± 0.99
NARS total	scores	2.43 ± 0.68	1.74 ± 0.33	2.64 ± 0.30	3.42 ± 0.40

**Table 4:** Adjusted odds ratios related to members of Profiles 2 and 3 compared with Profile 1.

Variables	Categories	Profile 2 versus Profile 1 (Neutral vs. Optimistic)			Profile 3 versus Profile 1 (Ambivalent vs. Optimistic)		
		OR	95 CI for OR	*p*	OR	95 CI for OR	*p*
Age	<45 years old	0.475	0.148–1.529	0.212	0.158	0.045–0.551	0.004 *
	45–59 years old	1.283	0.393–4.191	0.680	0.210	0.058–0.758	0.017 *
	≥60 years old	Ref	Ref	Ref	Ref	Ref	Ref
Education level	Junior high school or below	9.211	2.603–32.594	0.001 *	31.198	7.547–128.971	<0.001 *
	High school	4.599	1.388–15.239	0.013 *	11.378	2.638–49.074	0.001 *
	Junior college	3.967	1.717–9.166	0.001 *	2.993	0.889–10.075	0.077
	Bachelor’s degree or above	Ref	Ref	Ref	Ref	Ref	Ref
Monthly income	≤5000 CNY (≤$745)	0.420	0.206–0.857	0.017 *	0.456	0.171–1.218	0.117
	>5000 CNY (>$745)	Ref	Ref	Ref	Ref	Ref	Ref
Chronic diseases or not	Yes	1.357	0.648–2.839	0.418	0.733	0.273–1.964	0.537
	No	Ref	Ref	Ref	Ref	Ref	Ref
Experience with computers	Yes	3.516	1.260–9.811	0.016 *	2.693	0.847–8.559	0.093
	No	Ref	Ref	Ref	Ref	Ref	Ref
Experience with wearable devices	Yes	0.850	0.492–1.469	0.560	0.208	0.083–0.523	0.001 *
	No	Ref	Ref	Ref	Ref	Ref	Ref
Experience with automatic devices	Yes	1.170	0.563–2.432	0.675	0.460	0.192–1.103	0.082
	No	Ref	Ref	Ref	Ref	Ref	Ref
Whether they follow robot-related news or not	Never	5.176	1.823–14.695	0.002 *	0.779	0.191–3.170	0.727
	Sometimes	6.275	3.262–12.068	<0.001 *	1.468	0.567–3.801	0.429
	Always	Ref	Ref	Ref	Ref	Ref	Ref

Note: OR = odds ratio; CI = confidence interval; Ref = reference category; * = Statistically significant.

## Data Availability

The data presented in this study are available on request from the corresponding author.
